# Dietary Glycemic Load and Glycemic Index and Risk of Cerebrovascular Disease in the EPICOR Cohort

**DOI:** 10.1371/journal.pone.0062625

**Published:** 2013-05-23

**Authors:** Sabina Sieri, Furio Brighenti, Claudia Agnoli, Sara Grioni, Giovanna Masala, Benedetta Bendinelli, Carlotta Sacerdote, Fulvio Ricceri, Rosario Tumino, Maria Concetta Giurdanella, Valeria Pala, Franco Berrino, Amalia Mattiello, Paolo Chiodini, Salvatore Panico, Vittorio Krogh

**Affiliations:** 1 Fondazione IRCCS Istituto Nazionale dei Tumori, Milan, Italy; 2 Department of Public Health, University of Parma, Parma, Italy; 3 Molecular and Nutritional Epidemiology Unit, ISPO-Cancer Research and Prevention Institute, Florence, Italy; 4 Center for Cancer Prevention (CPO-Piemonte), Turin, Italy; 5 Human Genetics Foundation (HuGeF), Turin, Italy; 6 Cancer Registry and Histopathology Unit Department of Oncology, Ospedale Civile “M.P. Arezzo”, Ragusa, Italy; 7 Cancer Registry Department of Oncology, Ospedale Civile “M.P. Arezzo”, Ragusa, Italy; 8 Department of Clinical and Experimental Medicine, University of Naples Federico II, Naples, Italy; 9 Department of Public Health and Preventive Medicine, University II of Naples, Italy; Innsbruck Medical University, Austria

## Abstract

**Background:**

Studies on the association of stroke risk to dietary glycemic index (GI) and glycemic load (GL) have produced contrasting results.

**Objective:**

To investigate the relation of dietary GI and GL to stroke risk in the large EPIC-Italy cohort (EPICOR) recruited from widely dispersed geographic areas of Italy.

**Design:**

We studied 44099 participants (13,646 men and 30,453 women) who completed a dietary questionnaire. Multivariable Cox modeling estimated adjusted hazard ratios (HRs) of stroke with 95% confidence intervals (95%CI). Over 11 years of follow-up, 355 stroke cases (195 ischemic and 83 hemorrhagic) were identified.

**Results:**

Increasing carbohydrate intake was associated with increasing stroke risk (HR = 2.01, 95%CI = 1.04–3.86 highest vs. lowest quintile; p for trend 0.025). Increasing carbohydrate intake from high-GI foods was also significantly associated with increasing stroke risk (HR 1.87, 95%CI = 1.16–3.02 highest vs. lowest, p trend 0.008), while increasing carbohydrate intake from low-GI foods was not. Increasing GL was associated with significantly increasing stroke risk (HR 2.21, 95%CI = 1.16–4.20, highest vs. lowest; p trend 0.015). Dietary carbohydrate from high GI foods was associated with increased both ischemic stroke risk (highest vs. lowest HR 1.92, 95%CI = 1.01–3.66) and hemorrhagic stroke risk (highest vs. lowest HR 3.14, 95%CI = 1.09–9.04). GL was associated with increased both ischemic and hemorrhagic stroke risk (HR 1.44, 95%CI = 1.09–1.92 and HR 1.56, 95%CI = 1.01–2.41 respectively, continuous variable).

**Conclusions:**

In this Italian cohort, high dietary GL and carbohydrate from high GI foods consumption increase overall risk of stroke.

## Introduction

Hyperglycemia, insulin resistance and associated disorders of lipid metabolism (dyslipidemias) are known to increase the risk of cardiovascular disease (CVD) and are also known to be caused, at least in part, by diet [1]. High carbohydrate diets seem not only to promote hyperglycemia, but also to raise fasting triacylglycerol levels and reduce levels of high-density lipoprotein (HDL) cholesterol [2]. Postprandial hyperglycemia is emerging as an important and independent risk factor for stroke [3] from which it follows that a high carbohydrate diet is likely to increase the risk of stroke. However, carbohydrates vary markedly in physical form, chemical structure, particle size, and fiber content, and also in their ability to increase postprandial blood glucose levels. The glycemic index (GI) of a food is a commonly used marker of postprandial blood glucose response [4]. Given that the amount of carbohydrate in a food (or overall diet) can vary and have a variable influence on the postprandial glycemic response, the glycemic load (GL) (product of the GI of a food item and its available carbohydrate content) is also used as an estimator of the overall glycemic effect of the diet.

Associations of dietary GI and GL with risk of stroke have been assessed in various populations. Two of six prospective studies reported higher risk of stroke [5] or death from stroke [6] in persons with high dietary GI. Other studies found associations in one or more subgroups; thus a positive association between GI and risk of stroke was reported in men [7], while both GI and GL were associated with increased stroke risk in overweight women [8]. Furthermore risk has been found to vary with type of stroke. Two studies found that carbohydrate and GL were associated with increased risk of hemorrhagic stroke but not of ischemic stroke [5], [9].

We have previously investigated the association of GL and GI with risk of coronary heart disease (CHD) in the Italian EPICOR cohort – a large cohort recruited from widely dispersed geographic areas of Italy. We found that high dietary GL and high carbohydrate intake from high-GI foods increased the risk of CHD in women but not men [10]. In the present study we investigated the relation of dietary GL and GI to stroke in the same cohort.

## Subjects and Methods

EPICOR is a prospective collaborative investigation of the causes of cardiovascular diseases being performed on Italian volunteers recruited from 1993 to 1998 as part of the European Prospective Investigation into Cancer and Nutrition (EPIC) [11]. A total of 47,021 participants (14,863 men; 32,158 women, aged 35–75) were recruited to EPICOR from five centers: Varese (n = 11,809) and Turin (n = 10,528) in northern Italy; Florence (n = 13 436) in central Italy; and Ragusa (n = 6264) and Naples (n = 4984) in the South. Only women were recruited in Naples.

We excluded participants with prevalent CVD at baseline (n = 776); those who did not complete the dietary or lifestyle questionnaires (n = 907); and those in whom the ratio of total energy intake (determined from the questionnaire) to basal metabolic rate (determined by Harris-Benedict equation [12] at either extreme of the distribution (cutoffs first and last-half percentiles) (in order to reduce the impact of implausible extremes) (n = 794). We also excluded those stating they had diabetes and reported receiving medication for diabetes (n = 404). Thus, the analyses were performed on 44,099 participants with a mean follow-up of 10.9 years.

### Ethics Statement

The study protocol was approved by the ethics committees at the Human Genetics Foundation (Turin, Italy). At baseline, participants signed a written informed consent to use clinical data for research. All consent forms were stored with barcode ID for subject identification. The ethics committees approved this consent procedure.

### Baseline measurements

#### Diet

Dietary information was obtained using semi-quantitative food frequency questionnaires designed to capture local dietary habits during the previous year. Three FFQs were developed: one for the northern and central Italian centers of Varese, Turin and Florence, one for Ragusa, and one for Naples. The southern (Ragusa and Naples) questionnaires were administered by trained interviewers, whereas the north-central questionnaires were self-administered. All FFQs were validated [13]. Detailed descriptions of the FFQ are given elsewhere [14]. The food items were then linked, using specifically designed software [14], to Italian Food Tables [15] to obtain estimates of daily intake of 37 macro- and micro-nutrients plus energy. GIs of food items containing available carbohydrates were obtained from the Italian Glycemic Index Table (unpublished data, Brighenti F, PhD, Department of Public Health, University of Parma). This Table lists over 150 food items covering over 90% of the carbohydrate intake of people living in Italy, and includes all major Italian food items/preparations; it also contains recent data from the literature on the GIs of foods similar to those consumed in Italy. If there was no food item in the Table sufficiently similar to a given consumed item, GIs published elsewhere (International GI Tables [16] and www.glycemicindex.com.) and not specifically related to an Italian diet, were used. A detailed description of this Table is given elsewhere [10].

The average dietary GI for each participant was calculated as the sum of the GIs of each food item consumed, multiplied by the average daily amount consumed and the percentage of carbohydrate content, all divided by the total daily carbohydrate intake. The GL was calculated similarly except that there was no division by total carbohydrate intake. Each unit of GL is equivalent to the blood glucose-raising effect of consuming 1 g of glucose.

We divided carbohydrate intake into high- and low-GI foods, choosing a GI of 57 as the cutoff; we adopted this cutoff in order that high- and low-GI foods each contributed 50% of mean total carbohydrate intake in our cohort. The main sources of carbohydrates from high-GI foods in our cohort were bread (60.8%), sugar/honey and jam (9.1%), pizza (5.4%), and rice (3.2%); the main sources of carbohydrates from low-GI foods were pasta (33.3%), fruit (23.5%), and cakes (18.6%).

#### Lifestyle variables and anthropometric measurements

A standardized lifestyle questionnaire was completed by each participant; it was designed to obtain detailed information on reproductive history, alcohol consumption, smoking history, exposure to environmental tobacco smoke, medical history, physical activity and other lifestyle factors.

Weight and height were measured at enrolment according to the EPIC protocol, with participants in light clothing and shoes removed. Body mass index (BMI) was calculated as weight divided by height squared (kg/m^2^).

### Ascertainment and verification of major cerebrovascular disease

Record linkage between the EPICOR database and regional mortality and hospital discharge databases was performed after quality control of the EPICOR database. Suspected cerebrovascular disease (CBVD) deaths were identified from mortality files when International Classification of Diseases 10th edition codes I60–I69 were reported as an underlying cause of death or when codes E10–E14, I10–I15, I46, I49, and I70 were reported as an underlying cause in association with I60–I69. Fatal CBVD was assigned after verification against hospital discharge and clinical records. Persons with suspected CBVD were identified on hospital discharge forms through ICD9-CM codes 342, 430–434, or 436–438, or by procedure codes for carotid revascularization. Clinical records were always retrieved to verify CBVD. We used MONICA criteria to define CBVD [17]. Ischemic thrombotic stroke was diagnosed when brain infarction or similar was mentioned in the diagnosis and supported by computed tomography (CT) or magnetic resonance imaging (MRI) information. Hemorrhagic stroke was diagnosed when cerebral hemorrhage or similar was mentioned in the diagnosis and again supported by CT or MRI.

#### Follow-up

Subjects were followed up from study entry and until stroke diagnosis, death, emigration or until the end of the follow-up period, whichever occurred first. The date of the end of follow-up varied by center because hospital discharge file availability for updating varied as follows: December 31, 2003, for Florence; December 31, 2006, for Varese and Naples; December 31, 2007 for Ragusa; and December 31, 2008, for Turin.

### Statistical Analysis

Carbohydrate, carbohydrate from high GI- foods and carbohydrate from low GI- foods, starch, sugar, fiber intake, dietary GL and dietary GI were analyzed as categorical variables and adjusted for the energy intake using the regression-residual method [18]. Quintiles were defined on the whole cohort. The distributions of lifestyle and dietary baseline characteristics of study participants were assigned to quintiles of energy-adjusted GL. Multivariate Cox proportional hazard models were used to assess the association of these dietary components with stroke risk. Age was the primary time variable. All analyses were stratified by type of FFQ (north-central Italy, Naples and Ragusa) to control for differences in questionnaire design, and age at baseline (1-year categories). HRs with 95%CI were estimated for quintiles of dietary components (based on whole population), with the lowest quintile as reference. HRs were also calculated for 1 standard deviation increments of dietary variables as continuous variables.

Two models are presented: a ‘crude’ model (adjusted only for sex and stratified by type of questionnaire and age) and an adjusted model also adjusted for smoking (never smoker/former smoker/current smoker), years of education (<8 years/≥8 years), BMI (kg/m^2^), alcohol intake (g/day), non alcohol energy intake (kcal/day), physical activity (inactive, moderately inactive, moderately active, active), cereal fiber intake (g/day), saturated fat (g/day), monounsaturated fat (g/day), polyunsaturated fat (g/day). To test for trends, we determined the median of each quintile and modeled this as a continuous variable.

We tested the proportional hazard assumption for each food variable in relation to stroke risk using the method of Grambsch and Therneau [19]. In all cases, the proportional hazards assumption was satisfied (not shown).

To assess whether associations of stroke with total carbohydrate, GI and GL differed with sex or BMI, we stratified by sex and BMI, using product terms (0 and 1 for male and female, respectively, and for BMI ≤25 and >25, respectively) that were multiplied by the median value of the carbohydrate, GL or GI quintile to which the subject belonged. To assess the significance of interactions, we used a likelihood ratio test that compared the model that included the product term and the model that did not include it. STATA software (version 11.2; Stata Corp., TX) was used to perform the statistical analyses.

## Results

After a mean follow-up of 10.9 years, 355 participants were diagnosed as follows: 195 ischemic stroke and 83 hemorrhagic stroke, 42 carotid revascularizations, 31 death certificate only (DCO) cerebrovascular events and 4 unspecified types of stroke; 148 in men and 207 in women. Stroke incidence was highest in Varese (144 per 126,892 person-years), followed by Florence (80 cases per 104,464 person-years), Turin (78 cases per 124,392 person-years), Naples (25 cases per 55, 019 person-years) and Ragusa (28 cases per 69,581 person-years). **Table 1** shows the distribution of participant characteristic across quintiles of energy-adjusted dietary GL. Participants in the highest dietary GL quintile consumed less protein, saturated, monounsaturated, polyunsaturated fat and alcohol, higher intake of fiber from cereals and fruit and were more sedentary than those in the lowest GL quintile.

**Table 1:** Baseline distribution of nutrients and cardiovascular risk factors (standard deviation in parentheses) by quintiles of energy-adjusted glycemic load (GL) in EPICOR study.*

	Quintiles of energy-adjusted GL				
	I	II	III	IV	V
***N***	8824	8819	8814	8826	8816
***Overall glycemic index***	51.8(2.7)	52.6(2.5)	53.3(2.4)	54.1(2.34)	55.5(2.54)
***Glycemic load***	130.9(47.5)	132.1(45.3)	141.2(44.3)	157.8(45.3)	203.4(54.4)
***Protein*** (g/day)	108.5(30.5)	92.1(25.7)	87.4(25.2)	86.8(25.9)	93.8(26.2)
***Saturated Fat*** (g/day)	39.4(13.2)	31.7(10.4)	29.0(9.8)	27.7(9.8)	27.5(9.6)
***Monounsaturated Fat*** (g/day)	52.1(15.9)	42.6(12.6)	39.1(12.1)	37.4(12.1)	37.5(12.1)
***Polyunsaturated Fat*** (g/day)	12.5(4.9)	10.5(3.93)	9.9(3.7)	9.8(3.7)	10.5(3.8)
***Carbohydrate*** ** (g/day)**	252.7(90.9)	251.6(86.5)	265.7(84.3)	292.7(86.3)	367.0(99.1)
***High GI carbohydrate*** (g/day)	107.1(48.3)	112.3(47.6)	125.1(47.9)	148.2(51.3)	212.8(70.3)
***Low GI carbohydrate*** (g/day)	145.5(61.1)	139.3(57.5)	140.6(55.4)	144.4(56.7)	154.1(59.5)
***Starch*** (g/day)	148.5(67.7)	149.7(64.1)	161.6(64.0)	184.1 (67.6)	246.9 (82.0)
***Sugars*** (g/day)	103.9(42.7)	101.7(40.9)	103.8(40.6)	108.4 (42.1)	119.8(49.9)
***Fiber from potatoes***(g/day)	0.54(0.5)	0.44(0.4)	0.42(0.36)	0.42(0.36)	0.45(0.39)
***Fiber from vegetables***(g/day)	5.14(2.6)	4.34(2.1)	3.99(1.9)	3.80(1.8)	3.78(1.9)
***Fiber from legumes***(g/day)	1.76(1.7)	1.63(1.6)	1.62(1.6)	1.62(1.8)	1.74(1.9)
***Fiber from fruit*** (g/day)	6.84(4.5)	6.85(4.3)	7.18(4.6)	7.65(5.3)	8.77(6.5)
***Fiber form cereals*** (g/day)	7.4(4.4)	7.8(4.5)	8.7(5.0)	10.6(6.2)	15.8(10.1)
***Energy no alcohol*** (kcal/day)	2379(707)	2124(635)	2094(619)	2163(631)	2477(663)
***Alcohol*** (g/day)	22.7(22.8)	13.8(15.9)	10.7(13.6)	8.7(12.4)	7.14(11.1)
**Age** *(years)*	49.8(7.4)	50.2(7.6)	50.1(7.9)	50.1(7.8)	49.8(7.9)
**Body mass index** *(kg/m^2^)*	26.4(4.0)	26.0(4.1)	25.8(3.9)	25.7(4.1)	25.8(4.0)
***Total physical activity index*** (%)					
Sedentary	15.4	17.8	19.8	21.4	25.6
Moderately sedentary	20.7	21.6	20.7	20.0	17.0
Moderately active	22.5	20.4	20.0	19.3	17.8
Active	24.2	19.7	18.4	18.1	19.6
***Education*** (% ≥8 years)	19.8	19.9	20.2	20.1	21.0
***Smoking***					
Current (%)	21.0	18.8	19.2	19.2	21.8
Former (%)	22.6	20.1	19.1	19.2	19.0
Never (%)	17.9	20.6	21.0	20.9	19.6

* Unless otherwise indicated, data are expressed as mean (SD).

**Table 2** shows the associations of overall stroke risk with quintiles of energy-adjusted dietary carbohydrate, carbohydrate from high-GI foods, carbohydrate from low-GI foods, starch, sugar, dietary GL, dietary GI and fiber. Increasing intake of carbohydrate was associated with increasing risk of stroke, adjusted model, (HR = 2.01, 95%CI = 1.04–3.86 highest vs. lowest quintile; p for trend across quintiles 0.025). When carbohydrate intake was considered as a continuous variable, stroke hazard ratio for increasing carbohydrate intake was statistically significant (HR = 1.49, 95%CI = 1.18–1.90 for 1 standard deviation increments-adjusted model).

**Table 2:** Hazard ratios (with 95% confidence intervals) for stroke by increasing energy-adjusted quintiles of carbohydrate, high GI carbohydrate, low GI carbohydrate, starch, sugar, dietary glycemic index, glycemic load and fiber intake.*

	I	II	III	IV	V	P for trend	Intake as a continuous variable^ ç^
**Carbohydrate**							
N cases/N non-cases	85/8749	57/8749	68/8749	71/8749	74/8748		
Median (g/day)	232	266	287	307	339		
Crude HR (95% CI)^†^	1	0.72 (0.49–1.06)	0.88 (0.61–1.26)	0.93 (0.64–1.34)	1.18 (0.81–1.70)	0.313	1.09 (0.96–1.23)
Adjusted HR (95% CI)^$^	1	0.95 (0.62–1.45)	1.30 (0.81–2.08)	1.50 (0.87–2.57)	2.01 (1.04–3.86)	0.025	1.49 (1.18–1.90)
**High GI carbohydrate**							
N cases/N non-cases	72/8749	69/8749	69/8749	71/8749	74/8748		
Median (g/day)	86	116	137	160	203		
Crude HR (95% CI)^†^	1	0.93 (0.64–1.35)	0.90 (0.61–1.32)	1.08 (0.74–1.57)	1.48 (1.00–2.20)	0.037	1.14 (1.00–1.30)
Adjusted HR (95% CI)^$^	1	1.10 (0.74–1.63)	1.14 (0.75–1.73)	1.40 (0.91–2.16)	1.87 (1.16–3.02)	0.008	1.20 (1.03–1.41)
**Low GI carbohydrate**							
N cases/N non-cases	77/8749	58/8749	69/8749	75/8749	76/8748		
Median (g/day)	99	126	143	162	192		
Crude HR (95% CI)^†^	1	0.75 (0.51–1.10)	0.91 (0.63–1.31)	0.86 (0.59–1.26)	0.93 (0.63–1.35)	0.930	0.96 (0.84–1.09)
Adjusted HR (95% CI)^$^	1	0.86 (0.58–1.28)	1.09 (0.74–1.60)	1.03 (0.69–1.54)	1.01 (0.67–1.54)	0.731	0.98 (0.86–1.12)
**Starch**							
N cases/N non-cases	81/8749	64/8749	70/8749	68/8749	72/8748		
Median (g/day)	121	154	176	199	239		
Crude HR (95% CI)^†^	1	0.85 (0.59–1.21)	0.82 (0.57–1.19)	0.89 (0.61–1.29)	1.13 (0.77–1.66)	0.657	1.03 (0.90–1.17)
Adjusted HR (95% CI)^$^	1	0.99 (0.67–1.46)	1.06 (0.68–1.65)	1.21 (0.74–1.98)	1.50 (0.85–2.67)	0.151	1.17 (0.96–1.41)
**Sugar**							
N cases/N non-cases	77/8749	64/8749	70/8749	59/8749	85/8748		
Median (g/day)	69	90	104	120	150		
Crude HR (95% CI)^†^	1	1.00 (0.68–1.47)	0.91 (0.61–1.35)	0.83 (0.56–1.25)	1.31 (0.90–1.90)	0.161	1.07 (0.95–1.21)
Adjusted HR (95% CI)^$^	1	1.16 (0.78–1.73)	1.09 (0.72–1.64)	0.99 (0.65–1.53)	1.42 (0.93–2.16)	0.156	1.06 (0.93–1.21)
**Glycemic index**							
N cases/N non-cases	74/8749	64/8749	77/8749	75/8749	65/8748		
Median	50.0	52.0	53.4	54.8	56.9		
Crude HR (95% CI)^†^	1	0.92 (0.62–1.36)	1.17 (0.80–1.69)	1.30 (0.90–1.87)	1.03 (0.69–1.52)	0.417	1.09 (0.97–1.23)
Adjusted HR (95% CI)^$^	1	0.91 (0.61–1.36)	1.16 (0.79–1.70)	1.29 (0.88–1.89)	1.04 (0.68–1.58)	0.427	1.10 (0.96–1.26)
**Glycemic load**							
N cases/N non-cases	75/8749	70/8749	65/8749	77/8749	68/8748		
Median	121	140	153	165	186		
Crude HR (95% CI)^†^	1	1.06 (0.73–1.53)	0.91 (0.62–1.33)	1.15 (0.79–1.67)	1.27 (0.86–1.88)	0.225	1.11 (0.97–1.26)
Adjusted HR (95% CI)^$^	1	1.42 (0.94–2.15)	1.36 (0.84–2.20)	1.95 (1.14–3.33)	2.21 (1.16–4.20)	0.015	1.44 (1.16–1.77)
**Fiber**							
N cases/N non-cases	94/8749	67/8749	61/8749	71/8749	62/8748		
Median (g/day)	16.1	19.8	22.5	25.8	34.3		
Crude HR (95% CI)^†^	1	0.61 (0.43–0.89)	0.59 (0.41–0.86)	0.66 (0.46–0.96)	0.72 (0.46–1.13)	0.196	0.92 (0.77–1.09)
Adjusted HR (95% CI)^$ #^	1	0.72 (0.49–1.06)	0.73 (0.49–1.09)	0.83 (0.55–1.24)	0.87 (0.53–1.44)	0.802	0.99 (0.83–1.18)

* energy adjusted by residual method; †Adjusted for sex and stratified by FFQ and age;$ also adjusted for education, smoking, body mass index, alcohol, non alcohol energy intake, cereal fiber intake, saturated fat, monounsaturated fat, polyunsaturated fat and physical activity; # not adjusted for cereal fiber; ^ç^ for 1 SD increase.

Increasing intake of carbohydrate from high-GI foods was also significantly associated with increasing risk of stroke, adjusted model (HR 1.87, 95%CI = 1.16–3.02 highest vs. lowest quintile, p for trend across quintiles 0.008), while increasing the intake of carbohydrate from low-GI foods was not. Carbohydrate from high-GI foods intake was also associated with increased risk in the continuous model (HR = 1.20, 95%CI = 1.03–1.41 for 1 standard deviation increments-adjusted model). No significant association of starch and sugar intake with stroke risk was found.

GI had no significant influence on risk of stroke; whereas the forth (HR 1.95, 95%CI = 1.14–3.33) and fifth quintiles (HR 2.21, 95%CI = 1.16–4.20) of GL were associated with significantly higher stroke risk than the lowest quintile with p for trend across quintiles of 0.015. Increasing GL intake was also associated with increased risk in the continuous model (HR 1.44, 95%CI = 1.16–1.77 for 1 standard deviation increments-adjusted model).

Increasing fiber intake tended to be associated with lowered stroke risk but the reductions were not significant.

**Table 3** shows the associations of ischemic stroke with the same energy-adjusted variables as in Table 2. Increasing carbohydrate from high-GI foods consumption was associated with increasing risk of ischemic stroke in the adjusted model with p trend across quintiles 0.044 and only the highest quintile had an HR significantly above reference: adjusted model (HR 1.92, 95%CI = 1.01–3.66). Carbohydrate from high-GI foods was also significantly associated with increased ischemic stroke risk in the continuous model (HR 1.24, 95%CI = 1.01–1.53 for 1 standard deviation increments-adjusted model).

**Table 3:** Hazard ratios (with 95% confidence intervals) for ischemic stroke by increasing energy-adjusted quintiles of carbohydrate, high GI carbohydrate, low GI carbohydrate, starch, sugar, dietary glycemic index, glycemic load and fiber intake.*

	I	II	III	IV	V	P for trend	Intake as a continuous variable ^ç^
**Carbohydrate**							
N cases/N non-cases	51/8749	29/8749	34/8749	44/8749	37/8748		
Crude HR (95% CI) ^†^	1	0.55 (0.33–0.92)	0.63 (0.38–1.04)	0.84 (0.52–1.35)	0.80 (0.48–1.33)	0.582	0.95 (0.80–1.12)
Adjusted HR (95% CI)^$^	1	0.80 (0.45–1.43)	1.06 (0.56–2.02)	1.58 (0.77–3.21)	1.70 (0.70–4.10)	0.152	1.37 (0.99–1.88)
**High GI carbohydrate**							
N cases/N non-cases	43/8749	36/8749	39/8749	36/8749	41/8748		
Crude HR (95% CI) ^†^	1	0.77 (0.47–1.28)	0.78 (047–1.31)	0.83 (0.50–1.39)	1.18 (0.70–1.99)	0.539	1.06 (0.89–1.27)
Adjusted HR (95% CI)^$^	1	1.03 (0.61–1.75)	1.16 (0.66–2.02)	1.31 (0.73–2.36)	1.92 (1.01–3.66)	0.044	1.24 (1.01–1.53)
**Low GI carbohydrate**							
N cases/N non-cases	49/8749	29/8749	34/8749	41/8749	42/8748		
Crude HR (95% CI) ^†^	1	0.67 (0.40–1.12)	0.74 (0.45–1.22)	0.82 (0.49–1.35)	0.77 (0.46–1.29)	0.495	0.88 (0.74–1.05)
Adjusted HR (95% CI)^$^	1	0.81 (0.48–1.37)	0.93 (0.55–1.57)	1.04 (0.61–1.78)	0.89 (0.50–1.57)	0.912	0.93 (0.77–1.11)
**Starch**							
N cases/N non-cases	45/8749	36/8749	35/8749	42/8749	37/8748		
Crude HR (95% CI) ^†^	1	0.90 (0.56–1.45)	0.67 (0.40–1.13)	0.94 (0.58–1.53)	0.87 (0.51–1.50)	0.627	0.94 (0.79–1.13)
Adjusted HR (95% CI)^$^	1	1.29 (0.77–2.17)	1.17 (0.63–2.17)	1.93 (1.00–3.71)	1.95 (0.88–4.31)	0.072	1.25 (0.97–1.62)
**Sugar**							
N cases/N non-cases	43/8749	41/8749	36/8749	32/8749	43/8748		
Median							
Crude HR (95% CI) ^†^	1	1.02 (0.62–1.69)	0.75 (0.43–1.30)	0.84 (0.49–1.42)	1.11 (0.67–1.84)	0.789	1.01 (0.85–1.19)
Adjusted HR (95% CI)^$^	1	1.22 (0.73–2.04)	0.90 (0.51–1.58)	0.96 (0.55–1.70)	1.09 (0.61–1.94)	0.958	0.97 (0.81–1.17)
**Glycemic index**							
N cases/N non-cases	39/8749	33/8749	51/8749	37/8749	35/8748		
Crude HR (95% CI) ^†^	1	0.89 (0.51–1.55)	1.45 (0.88–2.40)	1.28 (0.76–2.14)	1.04 (0.60–1.80)	0.533	1.10 (0.93–1.30)
Adjusted HR (95% CI)^$^	1	0.88 (0.50–1.55)	1.48 (0.89–2.47)	1.36 (0.80–2.31)	1.14 (0.64–2.05)	0.331	1.16 (0.96–1.39)
**Glycemic load**							
N cases/N non-cases	46/8749	33/8749	38/8749	42/8749	36/8748		
Crude HR (95% CI) ^†^	1	0.77 (0.47–1.26)	0.73 (0.44–1.21)	0.91 (0.55–1.48)	0.89 (0.53–1.51)	0.775	0.99 (0.83–1.17)
Adjusted HR (95% CI)^$^	1	1.17 (0.67–2.04)	1.29 (0.68–2.43)	1.89 (0.93–3.86)	2.02 (0.86–4.78)	0.079	1.44 (1.09–1.92)
**Fiber**							
N cases/N non-cases	62/8749	34/8749	31/8749	37/8749	31/8748		
Crude HR (95% CI)^†^	1	0.43 (0.26–0.72)	0.46 (0.28–0.76)	0.53 (0.32–0.86)	0.46 (0.24–0.88)	0.024	0.80 (0.63–1.01)
Adjusted HR (95% CI) ^$^^#^	1	0.52 (0.31–0.89)	0.59 (0.34–1.02)	0.70 (0.40–1.21)	0.60 (0.30–1.23)	0.284	0.90 (0.71–1.14)

* energy adjusted by residual method; †Adjusted for sex and stratified by FFQ and age;$ also adjusted for education, smoking, body mass index, alcohol, non alcohol energy intake, cereal fiber intake, saturated fat, monounsaturated fat, polyunsaturated fat and physical activity; # not adjusted for cereal fiber; ^ç^ for 1 SD increase.

Increasing GL intake was associated with increased risk of ischemic stroke only in the continuous model (HR for 1standard deviation increments, 1.44, 95%CI = 1.09–1.92- adjusted model).

Increasing fiber intake was associated with decreasing ischemic stroke risk in the crude model but after adjustment for the other covariates risk reduction remained only in the second quintile and was no longer significant in the others.

**Table 4** shows the associations of hemorrhagic stroke risk with the same energy-adjusted variables as in Table 2. Carbohydrate and GL intake were significantly associated with increased ischemic stroke risk only in the continuous model (HR for 1 standard deviation increments 1.73, 95%CI = 1.04–2.87 and HR 1.56, 95%CI = 1.01–2.41, respectively- adjusted model).

**Table 4:** Hazard ratios (with 95% confidence intervals) for hemorrhagic stroke by increasing energy-adjusted quintiles of carbohydrate, high GI carbohydrate, low GI carbohydrate, starch, sugar, dietary glycemic index, glycemic load and fiber intake.*

	I	II	III	IV	V	P for trend	Intake as a continuous variable ^ç^
**Carbohydrate**							
N cases/N non-cases	19/8749	14/8749	16/8749	16/8749	18/8748		
Crude HR (95% CI)^†^	1	0.87 (0.40–1.86)	1.01 (0.47–2.13)	1.01 (0.47–2.17)	1.51 (0.72–3.16)	0.297	1.28 (0.99–1.66)
Adjusted HR (95% CI)^$^	1	0.87 (0.37–2.02)	1.05 (0.41–2.71)	1.04 (0.35–3.15)	1.40 (0.37–5.32)	0.628	1.73 (1.04–2.87)
**High GI carbohydrate**							
N cases/N non-cases	11/8749	22/8749	13/8749	20/8749	17/8748		
Crude HR (95% CI)^†^	1	2.12 (0.95–4.69)	1.07 (0.42–2.69)	2.24 (0.98–5.09)	2.62 (1.09–6.31)	0.043	1.24 (0.94–1.62)
Adjusted HR (95% CI)^$^	1	2.39 (1.04–5.50)	1.29 (0.48–3.46)	2.84 (1.12–7.23)	3.14 (1.09–9.04)	0.050	1.24 (0.89–1.72)
**Low GI carbohydrate**							
N cases/N non-cases	16/8749	15/8749	18/8749	17/8749	17/8748		
Crude HR (95% CI)^†^	1	0.66 (0.29–1.50)	1.01 (0.48–2.11)	0.83 (0.39–1.78)	0.84 (0.39–1.83)	0.829	1.06 (0.82–1.36)
Adjusted HR (95% CI)^$^	1	0.67 (0.29–1.55)	1.02 (0.48–2.18)	0.80 (0.36–1.78)	0.71 (0.30–1.66)	0.542	1.00 (0.76–1.33)
**Starch**							
N cases/N non-cases	22/8749	14/8749	16/8749	17/8749	14/8748		
Crude HR (95% CI)^†^	1	0.61 (0.29–1.28)	0.86 (0.43–1.71)	0.83 (0.40–1.72)	1.04 (0.47–2.31)	0.905	1.08 (0.82–1.42)
Adjusted HR (95% CI)^$^	1	0.50 (0.22–1.14)	0.65 (0.27–1.55)	0.60 (0.23–1.58)	0.61 (0.19–1.95)	0.464	0.98 (0.66–1.46)
**Sugar**							
N cases/N non-cases	14/8749	13/8749	18/8749	14/8749	24/8748		
Crude HR (95% CI)^†^	1	1.13 (0.48–2.64)	1.22 (0.53–2.80)	0.78 (0.31–1.96)	1.74 (0.79–3.80)	0.174	1.21 (0.97–1.51)
Adjusted HR (95% CI)^$^	1	1.18 (0.50–2.81)	1.35 (0.57–3.19)	0.87 (0.33–2.27)	1.83 (0.77–4.39)	0.195	1.23 (0.95–1.59)
**Glycemic index**							
N cases/N non-cases	13/8749	20/8749	14/8749	21/8749	15/8748		
Crude HR (95% CI)^†^	1	1.80 (0.82–3.93)	1.24 (0.53–2.94)	1.99 (0.90–4.36)	1.49 (0.64–3.49)	0.333	1.12 (0.88–1.43)
Adjusted HR (95% CI)^$^	1	1.79 (0.81–3.93)	1.25 (0.52–2.99)	2.00 (0.89–4.52)	1.55 (0.63–3.81)	0.321	1.13 (0.87–1.48)
**Glycemic load**							
N cases/N non-cases	15/8749	20/8749	15/8749	15/8749	18/8748		
Crude HR (95% CI)^†^	1	1.46 (0.70–3.08)	1.15 (0.51–2.57)	1.02 (0.44–2.40)	2.12 (0.98–4.61)	0.153	1.28 (0.99–1.66)
Adjusted HR (95% CI)^$^	1	1.59 (0.69–3.62)	1.33 (0.50–3.55)	1.28 (0.41–4.04)	2.58 (0.71–9.47)	0.219	1.56 (1.01–2.41)
**Fiber**							
N cases/N non-cases	12/8749	20/8749	16/8749	19/8749	16/8748		
Crude HR (95% CI)^†^	1	1.47 (0.66–3.27)	1.13 (0.48–2.65)	1.51 (0.66–3.46)	2.17 (0.87–5.41)	0.110	1.23 (0.88–1.71)
Adjusted HR (95% CI)^$ #^	1	1.53 (0.67–3.49)	1.17 (0.47–2.89)	1.48 (0.60–3.66)	1.87 (0.67–5.22)	0.292	1.12 (0.78–1.60)

* energy adjusted by residual method; †Adjusted for sex and stratified by FFQ and age;$ also adjusted for education, smoking, body mass index, alcohol, non alcohol energy intake, cereal fiber intake, saturated fat, monounsaturated fat, polyunsaturated fat and physical activity; # not adjusted for cereal fiber; ^ç^ for 1 SD increase.

Increasing carbohydrate consumption from high-GI foods was significantly associated with increasing hemorrhagic stroke risk (HR 3.14, 95%CI = 1.09–9.04 highest vs. lowest quintile, adjusted model), with a p for trend of 0.050. When carbohydrate from high-GI foods consumption was considered as a continuous variable, increasing intake was not statistically significant.

None of the other variables investigated, including carbohydrate intake from low-GI foods, was associated with hemorrhagic stroke risk. For all variables investigated, stroke risk did not differ significantly between men and women (tests for interaction were not significant), HRs were in the same direction and of similar size for both sexes (Data not shown). A similar lack of significant interaction was found for the high and low BMI categories (data not shown), however it is unlikely that the study has the statistical power to adequately examine such associations.

## Discussion

The present study is the first prospective study to find that high dietary GL is associated with significantly increased risk of overall stroke. By contrast dietary GI was not associated with stroke. The study also found that high carbohydrate intake was significantly associated with increased stroke risk – but on dividing carbohydrate into that derived from high GI foods and that from low GI foods, only carbohydrate from high GI foods was significantly associated with stroke risk. Thus, stroke risk depended not only on overall GL and quantity of carbohydrate intake, but also on the quality of carbohydrate consumed.

That increased stroke risk is associated with both high dietary GL and high carbohydrate from high GI foods is unsurprising, since total dietary GL is the sum of the products of each food's GI by its carbohydrate content. As its name suggests, therefore, dietary GL is a more sensitive measure of postprandial glycemia and insulin demand than dietary GI [20], [21]. And if stroke is related to the overall insulin demand of the diet, it is expected to be more strongly related to dietary GL than to dietary for GI, as we have observed.

Our findings are in agreement with those of two other studies, in which high dietary GL was non-significantly associated with increased risk of all types of stroke [8], [22]. In one of these, the Nurses' Health Study [8], stroke risk was significantly related to dietary GL only in overweight women. The other prospective investigations [5], [7], [9] found no association of dietary GL on overall stroke risk but two of them did find a weak association of GL with hemorrhagic stroke [5], [9]. Unlike previous studies, we found that dietary GL was associated with both ischemic and hemorrhagic stroke, however only when the variable was considered as continuous. Furthermore, we found that carbohydrate from high GI foods was significantly associated with risk of both ischemic and hemorrhagic stroke.

As noted, we found no significant association of GI with stroke. This is in contrast to previous findings that high dietary GI increased stroke risk in men [7] and in women [5], and also increased the risk of dying from stroke [6]. Oba et al. also reported that high GI increased the risk of ischemic stroke in women [5]. In two of these studies [5], [6] the mean energy-adjusted GI ranged from about 52 in the lowest to 70 in the highest quartile, while in our study the GI range was 52 to 55 indicating lower GI range in our Italian diet (data not shown) than in the diet of the Dutch and Japanese populations. In the other study [7], mean energy-adjusted GI across quartiles was not available.

There are likely to be several reasons why we have found consistent evidence of a relation between a high GL diet and increased risk of stroke, while other studies have found no or weak evidence for such an association.

Firstly, carbohydrate intake and dietary GL were high in our Mediterranean population, ranging form 252 g/day in the lowest to 355 g/day the highest quartile for carbohydrate and 131 in the lowest to 196 highest quartile for GL (data not shown), whereas in other studies [8], [9], [22] the corresponding figures were approximately 122 to 314 for carbohydrate and 79 to 175 g/day for GL. However, dietary differences in carbohydrate consumption and GL are unlikely to fully explain our results given that consumption was higher in a Japanese [5] study which found no influence of dietary carbohydrate in stroke risk.

Secondly, GL sources differed: in our population the major GL sources were white bread (36%), followed by pasta (13%); and when we divided carbohydrate according to GI, the major high GI source was white bread and major low GI food source was pasta. In other studies the major contributors to dietary GL were white bread and potatoes in Sweden (14.2%, 10.5% respectively) and the US (5.2%, 7.7% respectively) [23], [24] and rice in Japan [5] – all foods with high GI. Thus while in Italy the two main sources of GL had markedly different GIs permitting us to investigate a wide range of dietary GL; in the other countries it is likely that the range of dietary GL variation was much narrower.

We did not stratify the analyses by sex because there was no significant interaction between sex and carbohydrate, GL or GI; in fact HRs were in the same direction and of similar size for both sexes. Only two previous studies have presented analyses for men and women separately [5], [7], and although risk patterns related to dietary GI were reported to differ between the sexes, interactions were not significant. In a previous analysis of the same Italian EPICOR cohort [10], we found that high dietary GI, and high carbohydrate from high GI diet, were associated with significantly increased risk of coronary heart disease in women but not men, whereas in the present study the effect on stroke was the same in both sexes. Coronary heart disease and stroke share several [25] but not all risk factors, and how sex can influence the development of CHD and stroke differentially remains to be clarified.

All the carbohydrate-stroke associations found in this study support the hypothesis that high postprandial glycemia is the mechanism leading to increased stroke risk. Low GI carbohydrate sources such as whole grains and pasta are digested slowly so that the blood glucose peak is contained and the overall insulin response is low. By contrast consumption of high GI foods such as white bread results in a rapid and marked blood glucose peak that elicits a marked insulin response. Persistently high blood glucose and insulin as a result of a diet high in high GI carbohydrate may cause metabolic abnormalities giving rise to dyslipidemia that in turn increase the risk of stroke risk. Randomized trials have shown that low-GI and low-GL diets affect plasma concentrations of LDL cholesterol [26], [27], HDL cholesterol [28], triglycerides [29], [30], markers of inflammation [29] and thrombosis [30], and insulin resistance [31], in ways expected to reduce the risk of cerebrovascular disease. However, the observed effect of dietary interventions have been relatively modest and have not been observed consistently across trials [9].

Strengths of our study are its prospective design, and small number of participants lost to follow-up, limiting the possibility of selection bias. Furthermore, the fact that associations strengthened after adjustment for several recognized risk factors for cerebrovascular disease reduces the possibility of residual confounding. Another strength, particularly in comparison to previously published cohort studies, is that we used GI values that had mostly been determined on Italian foods. Since the glucose response, and probably also insulin response of a food, varies with the variety (for example rice) or mode of manufacture/preparation (e.g. parboiled vs. non parboiled rice, boiled rice vs. risotto), measured GIs of local foods are likely to be more accurate than those derived from international food tables. With regard to the food frequency questionnaires, these were designed to quantify the food items and preparations typically eaten in specific regions of Italy, and also to estimate the carbohydrate content of the diet; but they were not designed to produce measures of dietary GI or GL. However, it was relatively simple to apply GI values to the food items consumed. A study by Liu et al. [23] on food-frequency questionnaires broadly similar to ours showed it was possible to accurately estimate dietary GI and GL from questionnaire responses.

Another limit of our study is that dietary exposure is based on a single dietary assessment in which participants were asked about eating habits over the preceding year. Some participants may have changed their diet during follow-up, giving rise to some misclassification of exposure which would have weakened diet-disease associations. Finally, people do not generally eat single foods, but meals in which the GI value of an individual food can vary widely depending on how it is combined with other components and it is not possible to take such interactions into account using a food-frequency questionnaire. However strong correlations have been found between observed GI and GI values of mixed meals calculated from their components [32].

To conclude, in this Italian cohort we have found that high dietary GL and high consumption of carbohydrate from high-GI foods significantly increases risk of all types of stroke. However further prospective studies on larger cohorts and sufficiently long follow-up, are required to clarify the association of carbohydrate with stroke subtypes.

## References

[1] Levitan EB, Cook NR, Stampfer MJ, Ridker PM, Rexrode KM, et al (2008) Dietary glycemic index, dietary glycemic load, blood lipids, and C-reactive protein. Metabolism 57(3): 437–443. 18249220 10.1016/j.metabol.2007.11.002 PMC2262400

[2] Aryangat AV, Gerich JE (2010) Type 2 diabetes: postprandial hyperglycemia and increased cardiovascular risk. Vasc. Health Risk Manag. 6: 145–155. 10.2147/vhrm.s8216 PMC2860446 20448799

[3] Levitan EB, Song Y, Ford ES, Liu S (2004) Is nondiabetic hyperglycemia a risk factor for cardiovascular disease? A meta-analysis of prospective studies. Arch.Intern.Med. 164(19): 2147–2155. 10.1001/archinte.164.19.2147 15505129

[4] Jenkins DJ, Wolever TM, Taylor RH, Barker H, Fielden H, et al (1981) Glycemic index of foods: a physiological basis for carbohydrate exchange. Am.J.Clin.Nutr. 34(3): 362–366. 10.1093/ajcn/34.3.362 6259925

[5] Oba S, Nagata C, Nakamura K, Fujii K, Kawachi T, et al (2010) Dietary glycemic index, glycemic load, and intake of carbohydrate and rice in relation to risk of mortality from stroke and its subtypes in Japanese men and women. Metabolism 59(11): 1574–1582. 20303126 10.1016/j.metabol.2010.02.004

[6] Kaushik S, Wang JJ, Wong TY, Flood V, Barclay A, et al (2009) Glycemic index, retinal vascular caliber, and stroke mortality. Stroke 40(1): 206–212. 18948616 10.1161/STROKEAHA.108.513812

[7] Burger KN, Beulens JW, Boer JM, Spijkerman AM, van der AD (2011) Dietary glycemic load and glycemic index and risk of coronary heart disease and stroke in Dutch men and women: the EPIC-MORGEN study. PLoS.One. 6(10): e25955. 10.1371/journal.pone.0025955 PMC3187822 21998729

[8] Oh K, Hu FB, Cho E, Rexrode KM, Stampfer MJ, et al (2005) Carbohydrate intake, glycemic index, glycemic load, and dietary fiber in relation to risk of stroke in women. Am.J.Epidemiol. 161(2): 161–169. 10.1093/aje/kwi026 15632266

[9] Levitan EB, Mittleman MA, Hakansson N, Wolk A (2007) Dietary glycemic index, dietary glycemic load, and cardiovascular disease in middle-aged and older Swedish men. Am.J.Clin.Nutr. 85(6): 1521–1526. 10.1093/ajcn/85.6.1521 PMC4355937 17556687

[10] Sieri S, Krogh V, Berrino F, Evangelista A, Agnoli C, et al (2010) Dietary glycemic load and index and risk of coronary heart disease in a large italian cohort: the EPICOR study. Arch.Intern.Med. 170(7): 640–647. 10.1001/archinternmed.2010.15 20386010

[11] Palli D, Berrino F, Vineis P, Tumino R, Panico S, et al (2003) A molecular epidemiology project on diet and cancer: the EPIC-Italy Prospective Study. Design and baseline characteristics of participants. Tumori 89(6): 586–593. 14870823 10.1177/030089160308900602

[12] Harris JA, Benedict FG (1918) A Biometric Study of Human Basal Metabolism. Proc.Natl.Acad.Sci U.S.A 4(12): 370–373. 16576330 10.1073/pnas.4.12.370 PMC1091498

[13] Pisani P, Faggiano F, Krogh V, Palli D, Vineis P, et al (1997) Relative validity and reproducibility of a food frequency dietary questionnaire for use in the Italian EPIC centres. Int J Epidemiol 26 Suppl 1S152–S160. 9126543 10.1093/ije/26.suppl_1.s152

[14] PalaV, Sieri S, Palli D, Salvini S, Berrino F, et al (2003) Diet in the Italian EPIC cohorts: presentation of data and methodological issues. Tumori 89(6): 594–607. 14870824 10.1177/030089160308900603

[15] Salvini S, Parpinel M, Gnagnarella P, Maisonneuve P, Turrini A (1998) Banca dati di composizione degli alimenti per studi epidemiologici in Italia. Milano: Istituto Europeo di Oncologia press.

[16] Atkinson FS, Foster-Powell K, Brand-Miller JC (2008) International tables of glycemic index and glycemic load values: 2008. Diabetes Care 31(12): 2281–2283. 18835944 10.2337/dc08-1239 PMC2584181

[17] Asplund K, Tuomilehto J, Stegmayr B, Wester PO, Tunstall-Pedoe H (1988) Diagnostic criteria and quality control of the registration of stroke events in the MONICA project. Acta Med.Scand.Suppl 728: 26–39. 10.1111/j.0954-6820.1988.tb05550.x 3202029

[18] Willett W, Stampfer MJ (1986) Total energy intake: implications for epidemiologic analyses. Am.J.Epidemiol. 124(1): 17–27. 10.1093/oxfordjournals.aje.a114366 3521261

[19] Grambsch PM, Therneau TM (1994) Proportional Hazards Tests and Diagnostics Based on Weighted Residuals. Biometrika 81: 515–526.

[20] Bao J, Atkinson F, Petocz P, Willett WC, Brand-Miller JC (2011) Prediction of postprandial glycemia and insulinemia in lean, young, healthy adults: glycemic load compared with carbohydrate content alone. Am.J.Clin.Nutr. 93(5): 984–996. 10.3945/ajcn.110.005033 21325437

[21] Roberts CK, Liu S (2009) Effects of glycemic load on metabolic health and type 2 diabetes mellitus. J.Diabetes Sci.Technol. 3(4): 697–704. 10.1177/193229680900300414 PMC2769963 20144316

[22] Beulens JW, de Bruijne LM, Stolk RP, Peeters PH, Bots ML, et al (2007) High dietary glycemic load and glycemic index increase risk of cardiovascular disease among middle-aged women: a population-based follow-up study. J.Am.Coll.Cardiol. 50(1): 14–21. 10.1016/j.jacc.2007.02.068 17601539

[23] Liu S, Manson JE, Stampfer MJ, Holmes MD, Hu FB, et al (2001) Dietary glycemic load assessed by food-frequency questionnaire in relation to plasma high-density-lipoprotein cholesterol and fasting plasma triacylglycerols in postmenopausal women. Am.J.Clin.Nutr. 73(3): 560–566. 10.1093/ajcn/73.3.560 11237932

[24] Levitan EB, Westgren CW, Liu S, Wolk A (2007) Reproducibility and validity of dietary glycemic index, dietary glycemic load, and total carbohydrate intake in 141 Swedish men. Am.J.Clin.Nutr. 85(2): 548–553. 10.1093/ajcn/85.2.548 17284756

[25] Rosamond W, Flegal K, Furie K, Go A, Greenlund K, et al (2008) Heart disease and stroke statistics – 2008 update: a report from the American Heart Association Statistics Committee and Stroke Statistics Subcommittee. Circulation 117(4): e25–146. 18086926 10.1161/CIRCULATIONAHA.107.187998

[26] McMillan-Price J, Petocz P, Atkinson F, O'neill K, Samman S, et al (2006) Comparison of 4 diets of varying glycemic load on weight loss and cardiovascular risk reduction in overweight and obese young adults: a randomized controlled trial. Arch.Intern.Med. 166(14): 1466–1475. 10.1001/archinte.166.14.1466 16864756

[27] Sloth B, Krog-Mikkelsen I, Flint A, Tetens I, Bjorck I, et al (2004) No difference in body weight decrease between a low-glycemic-index and a high-glycemic-index diet but reduced LDL cholesterol after 10-wk ad libitum intake of the low-glycemic-index diet. Am.J.Clin.Nutr. 80(2): 337–347. 10.1093/ajcn/80.2.337 15277154

[28] Jenkins DJ, Kendall CW, McKeown-Eyssen G, Josse RG, Silverberg J, et al (2008) Effect of a low-glycemic index or a high-cereal fiber diet on type 2 diabetes: a randomized trial. JAMA 300(23): 2742–2753. 19088352 10.1001/jama.2008.808

[29] Pereira MA, Swain J, Goldfine AB, Rifai N, Ludwig DS (2004) Effects of a low-glycemic load diet on resting energy expenditure and heart disease risk factors during weight loss. JAMA 292(20): 2482–2490. 15562127 10.1001/jama.292.20.2482

[30] Ebbeling CB, Leidig MM, Sinclair KB, Seger-Shippee LG, Feldman HA, et al (2005) Effects of an ad libitum low-glycemic load diet on cardiovascular disease risk factors in obese young adults. Am.J.Clin.Nutr. 81(5): 976–982. 10.1093/ajcn/81.5.976 15883418

[31] Brand-Miller J, Buyken AE (2012) The glycemic index issue. Curr.Opin.Lipidol. 23(1): 62–67. 10.1097/MOL.0b013e32834ec705 22157060

[32] Wolever TM, Yang M, Zeng XY, Atkinson F, Brand-Miller JC (2006) Food glycemic index, as given in glycemic index tables, is a significant determinant of glycemic responses elicited by composite breakfast meals. Am.J.Clin.Nutr. 83(6): 1306–1312. 10.1093/ajcn/83.6.1306 16762941

